# *ESR1* Fusions Invoke Breast Cancer Subtype-Dependent Enrichment of Ligand-Independent Oncogenic Signatures and Phenotypes

**DOI:** 10.1210/endocr/bqae111

**Published:** 2024-08-29

**Authors:** Megan E Yates, Hunter Waltermire, Kanako Mori, Zheqi Li, Yiting Li, Hannah Guzolik, Xiaosong Wang, Tiantong Liu, Jennifer M Atkinson, Jagmohan Hooda, Adrian V Lee, Steffi Oesterreich

**Affiliations:** Women’s Cancer Research Center, Magee-Women Research Institute, Pittsburgh, PA 15213, USA; UPMC Hillman Cancer Center, Pittsburgh, PA 15213, USA; Medical Scientist Training Program, University of Pittsburgh School of Medicine, Pittsburgh, PA 15213, USA; Integrative Systems Biology Program, University of Pittsburgh School of Medicine, Pittsburgh, PA 15213, USA; Women’s Cancer Research Center, Magee-Women Research Institute, Pittsburgh, PA 15213, USA; UPMC Hillman Cancer Center, Pittsburgh, PA 15213, USA; Biomedical Masters Program, University of Pittsburgh School of Medicine, Pittsburgh, PA 15213, USA; Women’s Cancer Research Center, Magee-Women Research Institute, Pittsburgh, PA 15213, USA; UPMC Hillman Cancer Center, Pittsburgh, PA 15213, USA; Physician Scientist Training Program, University of Pittsburgh School of Medicine, Pittsburgh, PA 15213, USA; Women’s Cancer Research Center, Magee-Women Research Institute, Pittsburgh, PA 15213, USA; UPMC Hillman Cancer Center, Pittsburgh, PA 15213, USA; Department of Medical Oncology, Dana-Farber Cancer Institute, Boston, MA 02215, USA; Women’s Cancer Research Center, Magee-Women Research Institute, Pittsburgh, PA 15213, USA; UPMC Hillman Cancer Center, Pittsburgh, PA 15213, USA; School of Medicine, Tsinghua University, Beijing, China; Women’s Cancer Research Center, Magee-Women Research Institute, Pittsburgh, PA 15213, USA; UPMC Hillman Cancer Center, Pittsburgh, PA 15213, USA; UPMC Hillman Cancer Center, Pittsburgh, PA 15213, USA; Department of Pathology, University of Pittsburgh School of Medicine, Pittsburgh, PA 15213, USA; Women’s Cancer Research Center, Magee-Women Research Institute, Pittsburgh, PA 15213, USA; UPMC Hillman Cancer Center, Pittsburgh, PA 15213, USA; School of Medicine, Tsinghua University, Beijing, China; Women’s Cancer Research Center, Magee-Women Research Institute, Pittsburgh, PA 15213, USA; UPMC Hillman Cancer Center, Pittsburgh, PA 15213, USA; Department of Pharmacology and Chemical Biology, University of Pittsburgh School of Medicine, Pittsburgh, PA 15213, USA; Women’s Cancer Research Center, Magee-Women Research Institute, Pittsburgh, PA 15213, USA; UPMC Hillman Cancer Center, Pittsburgh, PA 15213, USA; Women’s Cancer Research Center, Magee-Women Research Institute, Pittsburgh, PA 15213, USA; UPMC Hillman Cancer Center, Pittsburgh, PA 15213, USA; Integrative Systems Biology Program, University of Pittsburgh School of Medicine, Pittsburgh, PA 15213, USA; Department of Pharmacology and Chemical Biology, University of Pittsburgh School of Medicine, Pittsburgh, PA 15213, USA; Human Genetics Graduate Program, University of Pittsburgh School of Public Health, Pittsburgh, PA 15213, USA; Department of Human Genetics, University of Pittsburgh School of Public Health, Pittsburgh, PA 15213, USA; Women’s Cancer Research Center, Magee-Women Research Institute, Pittsburgh, PA 15213, USA; UPMC Hillman Cancer Center, Pittsburgh, PA 15213, USA; Integrative Systems Biology Program, University of Pittsburgh School of Medicine, Pittsburgh, PA 15213, USA; Department of Pharmacology and Chemical Biology, University of Pittsburgh School of Medicine, Pittsburgh, PA 15213, USA; Human Genetics Graduate Program, University of Pittsburgh School of Public Health, Pittsburgh, PA 15213, USA; Department of Human Genetics, University of Pittsburgh School of Public Health, Pittsburgh, PA 15213, USA

**Keywords:** breast cancer, endocrine resistance, estrogen receptor, fusion, metastasis, ILC

## Abstract

Breast cancer is a leading cause of female mortality and despite advancements in personalized therapeutics, metastatic disease largely remains incurable due to drug resistance. The estrogen receptor (ER, *ESR1*) is expressed in two-thirds of all breast cancer, and under endocrine stress, somatic *ESR1* mutations arise in approximately 30% of cases that result in endocrine resistance. We and others reported *ESR1* fusions as a mechanism of ER-mediated endocrine resistance. ER fusions, which retain the activation function 1- and DNA-binding domains, harbor *ESR1* exons 1 to 6 fused to an in-frame gene partner resulting in loss of the ER ligand-binding domain (LBD). We demonstrate that in a no-special type (invasive ductal carcinoma [IDC]-NST) and an invasive lobular carcinoma (ILC) cell line, ER fusions exhibit robust hyperactivation of canonical ER signaling pathways independent of estradiol or antiendocrine therapies. We employ cell line models stably overexpressing ER fusions with concurrent endogenous ER knockdown to minimize endogenous ER influence. Cell lines exhibited shared transcriptomic enrichment in pathways known to be drivers of metastatic disease, notably MYC signaling. Cells expressing the 3′ fusion partners *SOX9* and *YAP1* consistently demonstrated enhanced growth and cell survival. ILC cells expressing the *DAB2* fusion led to enhanced growth, survival, and migration, phenotypes not appreciated in the IDC-NST *DAB2* model. Herein, we report that cell line activity is subtype-, fusion-, and assay-specific, suggesting that LBD loss, the fusion partner, and the cellular landscape all influence fusion activities. Therefore, it will be critical to assess fusion frequency in the context of the clinicopathology.

**Significance**

ER fusion proteins exhibit diverse mechanisms to drive endocrine resistance in two breast cancer cell lines representing the no special type (invasive ductal carcinoma (IDC)-NST) and invasive lobular cancer (ILC) subtypes. Our insights into both the shared and unique mechanisms engaged by the ER fusions lays the foundation for further translational research and the development of new clinical management strategies.

Breast cancer comprises one-third of all malignancies occurring in women and is estimated to have resulted in a total of 43 700 US deaths in 2023 alone, statistics that reinforce breast cancer's status as a modern epidemic (1, 2). Recent advancement in tumor molecular subtyping has provided insights into oncological management and therapeutic intervention (3). A vast majority of breast cancers (70%) express the prototype oncogene estrogen receptor alpha (ERα, *ESR1*; herein denoted ER). Hormonal therapies, including aromatase inhibitors (ie, anastrozole and letrozole) and selective ER modulators (SERMs), notably tamoxifen, achieved a 40% reduction in mortality of primary breast cancer (4). The success of hormonal therapy, however, is limited; approximately 30% of early breast cancer cases develop de novo or acquired resistance, an additional 70% of patients with metastatic disease lack an appreciable treatment response (5-10). Identifying the key mechanisms of endocrine resistance remains an unmet clinical need.

Acquired endocrine resistance occurs through a multitude of adaptations, the most notable being genetic alterations in *ESR1*. Mutations in the ligand-binding domain (LBD) of ER induce a ligand-independent and constitutively active ER protein that is found enriched in metastatic, endocrine-resistant disease, resulting in poor prognosis (11, 12). Interestingly, ER loss is not believed to be a predominant mode of endocrine resistance as the majority of resistant tumors retain their ER positivity (13). In the last decade, enrichment of ER fusion proteins in metastatic tumors that have been treated with endocrine therapy have been identified as a novel mechanism of endocrine resistance (14, 15).

Fusion-associated carcinomas have been studied for more than half a century since the discovery of the recurrent Philadelphia chromosome in chronic myeloid leukemia (16). Identification of cancer-causal gene fusions has resulted in seminal clinical and pharmaceutical advancements, particularity in hematological malignancies, as well as a subset of solid tumors, including prostate and non–small cell lung cancer (16, 17). Recent studies from The Cancer Genome Atlas (TCGA) revealed an abundance of gene fusions in breast, ovarian, and stomach cancers (18). Gene fusion products resulting in constitutive oncogene activation or tumor suppressor inactivation have been described as primary drivers of numerous cancer types (17). A crucial caveat to gene fusion discovery, however, is the actionability of identified in-frame fusion transcripts and their subsequent potential translational implications (19).

We and others have reported gene fusion events involving *ESR1* in breast cancer, with one of the most common being a recurrent rearrangement between *ESR1* exons 1 and 2 and its neighboring gene, coiled-coil domain containing 170 (ESR1-CCDC170) (20, 21). Further studies of *ESR1* fusions in metastatic breast cancer unveiled recurrent *ESR1* fusions that consistently involve *ESR1* exons 1 to 6 (14, 22-24). Fused to heterogeneous 3′ partner genes, these *ESR1* fusions all retain *ESR1* exons 1 to 6, but lose their ER LBD, which we subsequently postulate to be ligand independent. Our targeted sequencing uncovered interchromosomal *ESR1* fusions to the disabled homologue 2 gene (chromosome 5p; ESR1-DAB2*)* identified in a lymph node metastasis, and to the SRY-box transcription factor 9 gene (chromosome 17q; ESR1-SOX9) in a metastatic liver lesion (14). Both fusions were discovered in patient tumors that had been previously treated with hormonal therapies. In addition, an ER fusion to the lipoma preferred partner (chromosome 3q; ESR1-LPP) was identified in a patient-derived xenograft model (Champions TumorGraft CTG-1350) developed from a metastatic liver lesion. An *ESR1* exon 6 ESR1-LPP fusion was also identified in Priestley and colleagues’ (25) whole-genome sequencing study analyzing metastatic solid tumors. An *ESR1* fusion to the Yes1-associated transcriptional regulator functional protein domains (chromosome 11q, ESR1-YAP1) was also detected in a patient-derived xenograft model (26). ESR1-DAB2, ESR1-SOX9, and ESR1-YAP1 overexpression in a cell line model revealed ligand-independent and endocrine-resistant fusion activity (14, 27). In the T47D no-special type (invasive ductal carcinoma [IDC]-NST) cell line, overexpression of the most significant *ESR1* fusion proteins revealed an estrogen response signature that was subsequently developed into a 24-gene signature as a biomarker for functional fusion activity (27). To date, this has been the only study to functionally characterize a set of identified *ESR1* fusion proteins, albeit using only one histological subtype of breast cancer. To further investigate these ER fusions, we engineered an IDC-NST and an invasive lobular carcinoma (ILC) model, each depleted of endogenous ER, to assess fusion functionality in different cellular backgrounds. Furthermore, we performed multiomic analyses of IDC-NST fusion-positive cells to further understand transcriptomic differences among fusions. Evaluation of *ESR1* fusion functionality in various cellular contexts begins to offer an understanding in how a 3′ fusion partner may affect the function of one of breast cancer's most prognostic and clinically actionable genes, *ESR1*.

## Materials and Methods

### Cell Culture

Cell lines employed in our research are sourced from American Type Culture Collection (ATCC): HEK293T (RRID: CVCL_0045), T47D (RRID: CVCL_0553), and MDA-MB-134-VI (RRID: CVCL_0617). All cells were maintained in media supplemented with 10% fetal bovine serum (FBS; Life Technologies No. 26140079): HEK293 in Dulbecco's modified Eagle Medium (DMEM, Life Technologies No. 11965118); T47D in Roswell Park Memorial Institute (RPMI, Life Technologies No. 11875-119) 1640, and MDA-MB-134-VI in a 50:50 composition of DMEM and Leibovitz's L-15 medium (Invitrogen No. 11415-114). MCF-7/C4-12 (RRID: CVCL_GX99) is an estrogen-independent MCF-7 (RRID: CVCL_0031) derivative generated by 9 months of continuous hormone deprivation (28). C4-12 cells were cultured in phenol-free minimum essential medium (MEM, Fisher No. A10488-01) alpha media supplemented with 10% charcoal stripped serum (CSS; Sigma-Aldrich No. F6765). Cell lines were maintained at 37 °C and 5% CO_2_. Cells were tested to be mycoplasma free and authenticated by the University of Arizona Genetics Core. Hormone deprivation (media change to improved MEM [IMEM, Fisher Scientific No. MT10026CV] supplemented with 10% CSS for 3 days) was performed for all experiments unless otherwise stated.

### Reagents

17β-Estradiol (E2), purchased from Sigma (No. E8875) and fulvestrant (ICI182,780) obtained from Tocris (No. 1047) were both reconstituted in 100% ethanol. Tamoxifen (4-hydroxytamoxifen, 4-OHT) was obtained from Sigma (No. H6278) and was reconstituted in dimethyl sulfoxide (DMSO; ATCC).

### Plasmid Construction and Stable Cell Line Generation

ER fusion plasmid constructs were engineered by Gene Universal by custom gene synthesis. Identified *ESR1* fusion sequences were supplied for integration into a pCDH-MSCV-MCS-EF1α-copGFP-T2A-Puro backbone vector obtained from Systems Biosciences (No. CD713B). The *ESR1* fusion sequence was inserted at NheI and SwaI restriction sites with an upstream Kozak sequence and human influenza hemagglutinin (HA) tag. A second ESR1-DAB2 plasmid in a pCDH-CMV-MCS-EF1-Puro (System Biosciences No. CD510B-1) was received as a kind gift from Dr Matthew Ellis (AstraZeneca; formal principal investigator at the Baylor College of Medicine, Texas). Lentiviral infection was employed for stable expression of ER chimeric plasmids in T47D, MDA-MB-134-VI, and C4-12 cells. Plasmids were transfected into HEK293T cells with packaging plasmids pMDL, pRev and pVSVG using PEI in a 3:1 PEI-to-DNA ratio. Incubated target media on packaging cells was collected and filtered onto target cell lines at approximately 70% confluency with polybrene at 36, 48, and 72 hours post transfection. Infected cells were selected with puromycin for at least 7 days.

### Short Hairpin *ESR1* Design and Infection

Short hairpin (sh) RNA targeting the 3′ untranslated region (UTR) of *ESR1* in exon 8 was originally inserted in the MLPE backbone (a kind gift from Scott W. Lowe, Memorial Sloan Kettering Cancer Center, New York): TGCTGTTGACAGTGAGCGACAGCAAGTTGATCTTAGTTAATAGTGAAGCCACAGATGTATTAACTAAGATCAACTTGCTGCTGCCTACTGCCTCGGA. MLPE-shESR1 and the plasmid for subcloning, SGEN (pRRL) (Dr Johannes Zuber, RRID:Addgene 111171), were digested at XhoI and EcoRI restriction sites. Gel purification using the Monarch DNA Gel Extraction Kit was performed to extract the shRNA sequence and column purification with the QIAprep PCR Purification Kit (Qiagen No. 28104) was performed to concentrate the digested SGEN plasmid. The purified shESR1 and SGEN DNA were digested with XhoI and EcoRI and subsequently ligated at a molar ratio of approximately 3:1 with 10× T4 DNA Ligase Buffer (New England BioLabs Inc No. B0202S) and T4 DNA Ligase (New England BioLabs Inc No. M020 M). An additional shRNA targeting the 3′ UTR was engineered by VectorBuilder (shESR1.2). Lentiviral infection was employed for stable expression of shESR1 in T47D and MDA-MB-134-VI cells. Infected cells were selected with Geneticin for at least 7 days.

### Small Interfering RNA Transient Transfection

T47D and MDA-MB-134-VI cell lines were plated at equal cell densities in 6-well plates to reach 70% confluency in a 24-hour period. Two custom short interfering (si) RNA oligos targeting *ESR1* were designed and ordered from Horizon Discovery/Dharmacon: UCUCUAGCCAGGCACAUUC (sense sequence, ESR1_1) and UCAUCGCAUUCCUUGCAAA (sense sequence, ESR1_2). siGENOME nontargeting siRNA was obtain for a scramble control (Horizon Discovery No. D-001206-14). Cells collected for immunoblotting were infected with siRNA for 72 hours before lysis. Cells infected with siRNA for ER response element (ERE)-luciferase reporter assaying were infected for 72 hours with siRNA before plate reading. Equivalent quantities of siESR1 or scramble siRNA (scRNA, nontargeting control [siNTC]) were forward transfected following the Lipofectamine RNAiMAX (Thermo Fisher Scientific No. 13778150) protocol according to the manufacturer's instructions.

### Immunoblotting

Cellular protein lysates were harvested using radioimmunoprecipitation assay buffer (50 mM Tris pH 7.4, 150 mM NaCl, 1 mM EDTA, 0.5% Nonidet P-40, 0.5% sodium deoxycholate, 0.1% SDS) supplemented with 1× HALT protease and phosphatase cocktail (Thermo Fisher No. 78442). Samples were vortexed, probe-sonicated for 15 seconds, and centrifuged at 14 000 rpm at 4 °C for 15 minutes. Protein concentration was assessed using the Pierce Bicinchoninic acid protein assay (Thermo Fisher No. 23225). Protein sample was run on a 10% sodium dodecyl sulfate–polyacrylamide gel electrophoresis gel followed with a transfer at 4°C for 90 minutes. Membranes were blocked for 1 hour with Intercept phosphate-buffered saline (PBS) blocking buffer (LiCor No. 927-40000) at room temperature with rocking. Antibody probing was performed overnight at 4 °C with rocking: ERα clone 60C (Millipore No. 04-820, RRID:AB_1587018); HA (C29F4) (Cell Signaling Technologies No. 3724, RRID:AB_1549585); and β-actin (Millipore Sigma No. A5441, RRID:AB_ 476744). After removal of primary antibodies, blots were washed 3 times with 1× PBS-Tween 20 (0.1%) for 15 minutes. Secondary antibodies were applied for a 1-hour room temperature incubation (1:10 000; antimouse 680LT [LiCor No. 925-68020, RRID:AB_10706161]; antirabbit 800CW [LiCor No. 925-32211, RRID:AB_2651127]). Imaging of membranes was performed on the LiCor Odyssey CLx Imaging system.

### Estrogen Receptor Response Element–Tk-Luc Reporter Assay

C4-12, T47D, and MDA-MB-134-VI cells were seeded in triplicate in 24-well plates and HEK293T cells were seeded in replicates of 6 in 96-well-plates. Seeding density was calculated to reach 70% confluency within 24 hours. At 24 hours post plating, cells were washed with 1× PBS before addition of hormone-deprivation media. Cells were cotransfected at a ratio of 5:1 with a vector harboring a firefly luciferase gene under an ERE promoter (ERE-Tk-Luc) and a vector harboring renilla luciferase under a null promoter using the Lipofectamine 3000 transfection method following the manufacturer's protocol. At 24 hours post transfection, cells were treated with either 1-nM E2, 1-nM E2 and 100-nM fulvestrant, 1-nM E2 and 100-nM tamoxifen, or equal volume of 100% ethanol. C4-12, T47D, and MDA-MB-134-VI cells were lysed 48 hours post transfection with 100 µL 1× passive lysis buffer (Promega No. E1960) at room temperature for 20 minutes. Following lysis, 20 µL of lysed cells were transferred to a white 96-well plate in duplicates before measurement using the Dual-Luciferase Reporter Assay (Promega No. E1960). HEK293T cells were lysed and assayed using the Dual-Glo kit following the manufacturer's protocol (Promega No. E2940). Luminescence was measured using a 2-injector dual system on the GLOMAX-Multi + Microplate Multimode Reader (Promega). Relative luminescence units were calculated by normalizing oxyluciferin readouts to corresponding renilla measurements and in the HEK293T cells an additional normalization to nontransfected control cells was performed.

### Immunofluorescence Staining

T47D shESR1 cells were plated at a density of 150 000 cells/well on glass coverslips in 24-well plates. At 24 hours post seeding, cells were fixed on ice with 4% paraformaldehyde for 30 minutes followed by blocking in blocking buffer (0.3% Triton X-100, 5% BSA, 1× Dulbecco's PBS) for 1 hour at room temperature. Primary antibody was incubated overnight at 4 °C: ER (1:50 dilution, Lecia Biosystems No. NCL-L-ER-6F11, RRID:AB_563706). Secondary antibody incubation was performed for 1 hour at room temperature: antimouse AlexaFluor647 (1:200 dilution, Thermo Fisher Scientific No. A28181, RRID:AB_2536165). Cells were nuclear-stained with Hoechst 33342 (1:10 000 dilution, proxy to DAPI stain, Thermo Fisher Scientific No. H3570) for 10 minutes. Coverslips were mounted with Aqua-Poly/Mount (Polysciences No. 18606-20) and slides were imaged on a Nikon A1 confocal microscope with the 40× objective.

### RNA Sequencing

Hormone-deprived T47D shESR1 cells were plated at equivalent cell densities in triplicate in 6-well plates to reach a 70% confluency at 24 hours. At 24 hours post seeding, cells were treated with 100 nM of ICI or equivalent volume of 100% ethanol for 24 hours. RNA extraction was performed using the Qiagen RNeasy Mini Kit (Qiagen No. 74106). Purified RNA samples had library preparation, quality control, and sequencing conducted at the UPMC Genome Center (Pittsburgh, Pennsylvania). Messenger RNA (mRNA) sequencing of paired-end 1 × 100 base pair (bp) reads was employed on a NovaSeq 6000 platform using a flow cell SP-200 to achieve a sequencing depth of 19 million reads per sample. RNA sequencing data have been deposited in NCBI's Gene Expression Omnibus and are accessible through GEO SuperSeries accession number GSE266408 and SubSeries accession number GSE249723 (29).

### RNA Sequencing Analysis

RNA sequencing quality assurance was run using FastQC v0.11.8 (30). Expression quantifications were performed using the Salmon v1.3.0 application (31) with reference index Ensemble GRCh38. RNA gene-level summarizations were generated by tximport v1.16.1 with the reference EnsDb.Hsapiens.v86 (32). Principal component analysis (PCA) was performed using the variance stabilizing transformation (VST) and plotPCA functions from the R package DESeq2 v1.30.1 (33). VST DESeq2 counts were used to observe unbiased hierarchical clustering using the R function pheatmap v1.0.12 (RRID:SCR_016418). Significantly differentially expressed genes (DEGs) were defined as genes with a *P* value of less than .05. No fold change threshold was applied. Venn diagram plots were generated using the VennDiagram package (34). Gene set enrichment analysis (GSEA) was performed using the R package fgsea v1.22.0 (35) with pathway lists derived using the msigdbr package (36) or CSEA was performed with the IndepthPathway package (37). The IndepthPathway package function input was normalized and count matrixes generated using the DESeq2 function. Disambiguation analyses used a *P* value of less than .01 (37). Data visualization was performed using the ggplot2 package (38). EstroGene (estrogene.org) is a curated collection of MicroArray analyses, RNA sequencing (RNAseq) analyses, and chromatin immunoprecipitation sequencing (ChIP-seq) analyses (39).

### Quantitative Real-Time Polymerase Chain Reaction

Hormone-deprived T47D shESR1 cells were seeded at 1 000 000 cells/well into 6-well plates. Cells were treated for 24 hours with either 1-nM estradiol or an equivalent volume of 100% ethanol. RNA was extracted from cells following the RNeasy Mini Kit protocol. Complementary DNA was synthesized using the iScript RT Supermix reagent (BioRad No. 1708890). quantitative real-time polymerase chain reaction (qRT-PCR) reactions were performed using the SybrGreen Supermix (BioRad No. 1726275). The ΔΔCt method was applied to analyze relative mRNA fold changes to an *RPLP0* internal control.

### Chromatin Immunoprecipitation Sequencing and Analysis

T47D shESR1 cells were plated at 80% to 90% confluency prior to hormone deprivation. Hormone-deprived cells were treated with 100% ethanol for 45 minutes. An additional hormone-deprived ESR1-WT plate was treated with 1-nM E2 for 45 minutes as an experimental positive control. Cells were fixed in 1% formaldehyde for 10 minutes at room temperature with rocking. The reaction was quenched with 0.125-M glycine for 5 minutes at room temperature with rocking. Post quenching, cells were washed 3 times in ice-cold 1× DPBS, followed by harvest in 1× DPBS supplemented with protease and phosphatase inhibitors at a 1:100 dilution. Harvested cells were centrifuged and cell pellets were resuspended in 1-mL Lysis Buffer 1 (50 mM Hepes-KOH (pH 7.5), 140 mM NaCl, 1 mM EDTA, 10% glycerol, 0.5% NP-40/Igepal CA-630, 0.25% Triton X-100) and rotated at 4 °C for 10 minutes to enrich for the nuclear fraction (40). Pelleted cells were resuspended in Lysis Buffer 2 (10-mM Tris-HCL [pH 8.0], 200-mM NaCl, 1-mM EDTA, 0.5-mM EGTA) and rotated for 5 minutes at 4 °C (40). Pelleted cells were resuspended in 300-µL Lysis Buffer 3 (10-mM Tris-HCL [pH 8.0], 100-mM NaCl, 1-mM EDTA, 0.5-mM EGTA, 0.1% sodium deoxycholate) (40) followed by 4 °C sonication for 10 cycles of 30-second pulses. Sonicated samples were then centrifuged at 14 000 rpm for 10 minutes at 4 °C. A small aliquot of supernatant was saved for input in ChIP sequencing. A total of 100-µL/sample of Protein A Dynabeads (Thermo Scientific No. 10001D) were incubated with either 0.66 µg of HA (Cell Signaling Technology No. 3724S, RRID:AB_1549585) or normal rabbit immunoglobulin G (Millipore No. 12-370, RRID:AB_145841) antibody for 10 minutes at 4 °C with rotation including the appropriate wash steps as described in the manufacturer's protocol. The cell lysate's supernatant protein concentration was measured using the Pierce BCA protein assay. A volume equivalent to 500 µg of protein in each supernatant was incubated with the Dynabeads overnight at 4 °C with rotation. Following incubation, the Dynabeads were washed 6 times with radioimmunoprecipitation assay buffer (150-mM NaCl, 10-mM Tris [pH 7.2], 0.1% SDS, 1% Triton X-100, 1% sodium deoxycholate) using a magnetic stand for separation of beads and buffer. The beads were then washed once with TE buffer (10-mM EDTA, 50-mM Tris-HCL [pH 7.4]). ChIP samples and inputs were decrosslinked with 200 µL of elution buffer (1% SDS, 0.1-M NaHCO_3_) overnight at 65 °C. DNA was purified using the QIAquick PCR purification kit following the manufacturer's protocol. ChIP qRT-PCR quality control for *GREB1* was performed. HA ChIP was performed 3 times independently. The first biological replicate of samples was sent to the Health Sciences Sequencing Core at Children's Hospital of Pittsburgh (HSSC) for TruSeq ChIP Library preparation (Illumina) using paired indexing. Qubit quantification and TapeStation HS D1000 was performed for library quantity control. Additional size fragment purification was performed by HSSC with the remaining biological replicates. DNA sequencing was performed on an Illumina NextSeq 2000 P3 flow cell 100 at 2 × 61 bp reads with approximately 80 million reads per sample (overall 1.2 billion reads). ChIP sequencing data have been deposited in NCBI's Gene Expression Omnibus and are accessible through GEO SuperSeries accession number GSE266408 and SubSeries accession number GSE266407 (29).

ChIP-seq reads were aligned to Hg38 reference genome assembly using Bowtie 2.0 (41), and peaks were called using MACS2.0 with a *q* value of less than 0.1 (42) and visualized with the WashU Browser (43). We used the Diffbind (44) package to identify differential binding sites based on genomic occupancy and analyze gained and lost sites between ESR1-WT and each fusion. Heat map of binding intensity on ESR1-WT + E2 sites were visualized by Seqplots. For gene annotation from gained binding sites, peaks were selected and annotated genes were inspected in a ± 100-kb range of all the peaks using Binding and Expression Target Analysis (BETA) (45). *ESR1* motif counts were calculated using FIMO (Find Individual Motif Occurrences) from MEME Suite 5.5 (46). Other public ChIP-seq data sets in the T47D cell line were downloaded from Cistrome db (47-49) using the BigWig format aligned to hg38. Specifically, ER ChIP-seq files were from GSM1858650, GSM1858652, and GSM3212400; ATAC-seq was from GSM2241148; FOXA1 ChIP-seq was from GSM1858654; H3K4me3 and H3K27ac ChIP-seq were from GSM2131188 and GSM1693025, respectively.

### Cell Growth Assay

Hormone-deprived T47D shESR1 and MDA-MB-134-VI shESR1 cells were seeded in replicates of 6 in two dimensional (2D) and three dimensional (3D) (ultra-low attachment (ULA)) 96-well-plates at a cell density of 6000 cells/well and 8000 cells/well, respectively. A day zero plate was seeded for 2D and 3D conditions with corresponding day 4, approximate day 7, and approximate day 14 plates for each treatment condition. At 24 hours post seeding, cells were treated with 1-nM estradiol, 100 nM ICI, a combination of both, or an equivalent volume of 100% ethanol. Plates were collected on day 0, day 4, at 1 week, and at 2 weeks and measured by CellTiter-Glo (Promega No. PR-G7573) following the manufacturer's protocol. Cell viability values were calculated by blank cell deductions and normalization to corresponding day zero readings.

### Colony Formation Assay

Hormone-deprived T47D shESR1 and MDA-MB-134-VI shESR1 cells were seeded in triplicate in 6-well plates at a cell density of 36 000 cells/well and 70 000 cells/well, respectively. Cells were monitored daily, and hormone deprivation medium was refreshed every 4 to 5 days. The assay was stopped when well-displayed multiple overlapping individual colonies were seen. Cells were fixed on ice with ice-cold 100% methanol and stained with 0.5% Crystal Violet in 40% methanol. Wells were imaged on an Olympus SZX16 dissecting microscope. Quantification was performed using the ImageJ threshold counting tool and particle analyzer plugin.

### Wound Scratch Assay

Hormone-deprived T47D shESR1 and MDA-MB-134-VI shESR1 cells were seeded in replicates of 8 onto Imagelock 96-well plates precoated with Matrigel (Corning No. 356237) at cell densities of 150 000 cells/well and 250 000 cells/well, respectively. At 24 hours post seeding, wounds were made to the middle of each well using a Wound Maker (Essen Bioscience No. 4493). T47D shESR1 cells were washed twice with 100-µL 1× PBS, and MDA-MB-134-VI shESR1 cells were washed once with 100-µL 1× PBS. Desired treatment, supplemented with 5-µg/mL Mitomycin C (Sigma Aldrich No. 10107409001), was added to cells. Cells were monitored and wound closure was imaged every 4 hours over the course of 72 hours using the IncuCyte Imaging system. Wound closure density was calculated using the IncuCyte system's wound scratch analysis module.

### Statistical Analysis

GraphPad Prism software version 7 and R version 4.0.0 were used for statistical analysis. All experimental results included biological replicates and were shown as a representative reading ± SEM. Two-way analysis of variance with post hoc Dunnett's multiple comparisons test was used showing enrichment in comparison to corresponding ESR1-WT treatment (#) or to intraconstruct vehicle treatment (*) in each ERE-luciferase reporter assay, wound scratch, and qPCR assay. An unpaired *t* test was used for colony formation assays. */#*P* less than or equal to .05; **/##*P* less than or equal to .01; ***/###*P* less than or equal to .001; and ****/####*P* less than .0001.

## Results

### In Vitro Models Show *ESR1* Fusions Discovered in Metastatic Breast Cancer Are Hyperactive and Ligand-Independent When Overexpressed

**Figure 1.**
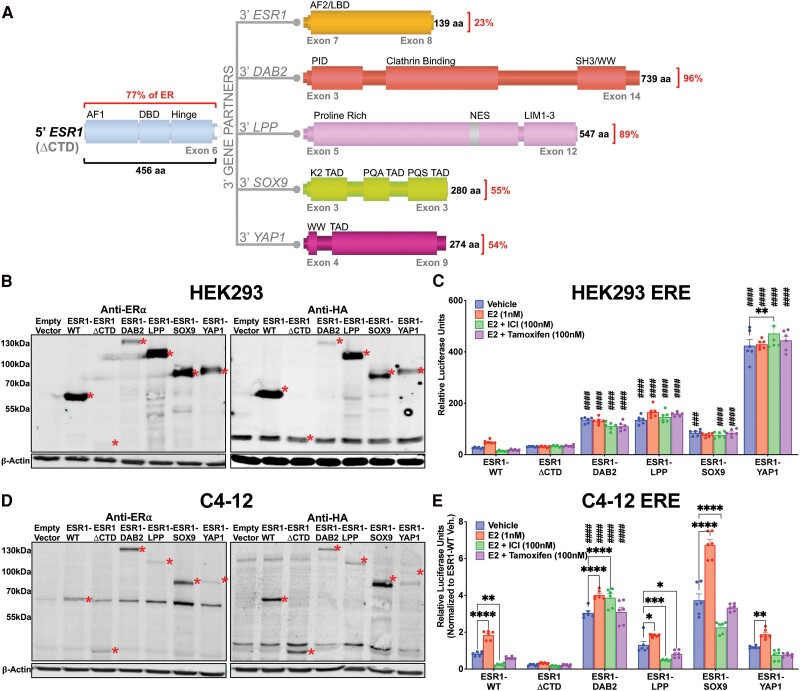
Estrogen receptor (ER) fusions are hyperactive in a ligand-resistant manner. A, Schematic of ER fusions. Percentages denote quantity of protein fused to the 5′ portion of ER (77% of the ER protein contained within exons 1-6 without C” terminal domain [ΔCTD]). Activation function (AF), DNA-binding domain (DBD), ligand-binding domain (LBD), phosphotyrosine interaction domain (PID), SRC homology 3-binding domain (SH3), Rsp5 or WWP domain (WW), nuclear export sequence (NES), LIM zinc-binding domain, transcriptional activation domain (TAD), proline-, glutamine-, and alanine-rich (PQA), proline-, glutamine-, and serine-rich (PQS), substrate-binding region (Sub), and glycogen synthase 1 interaction region (GYS1). B, Anti-ER and anti-HA immunoblots of transiently overexpressed HA-tagged wild-type ER (ESR1-WT), HA-tagged truncated ER (ESR1-ΔCTD) and HA-tagged ER fusion proteins in HEK293T cells. C, ERE-luciferase reporter assay in HEK293T cells transiently expressing ER constructs. Raw luminescence values normalized to internal control renilla luminescence. Representative experiment shown with bar graph equivalent to readings ± SEM, n = 2. D, Anti-ER and anti-HA immunoblots of stably overexpressed ESR1-WT, ESR1-ΔCTD, and ER fusion proteins in C4-12 cell line. Construct proteins denoted by red asterisks in all blots. β-Actin serves as loading control in all blots. E, ERE-luciferase reporter assay in C4-12 cells stably expressing ER constructs. Raw luminescence values normalized to internal control renilla luminescence and ESR1-WT vehicle activity. Representative experiment shown with bar graph equivalent to readings ± SEM, n = 3. Two-way analysis of variance with post hoc Dunnett's multiple comparisons test showing enrichment in comparison to corresponding ESR1-WT treatment (#) or to intra-construct vehicle treatment (*) in each assay. */#*P* less than or equal to .05; **/##*P* less than or equal to .01; ***/###*P* less than or equal to .001; ****/####*P* less than .0001.

Limited previous studies have discovered (Fig. 1A) and subsequently analyzed ER fusion proteins (Supplementary Fig. S1) (27, 49, 50). Transient expression of the *ESR1* fusion plasmids in human embryonic kidney HEK293 cells demonstrated expression of protein products at anticipated ER wild-type (ESR1-WT), ER truncation (*ESR1* exons 1-6 without ER C” terminal domain [ΔCTD]), or fusion protein size, detected by both anti-ERα and anti-HA antibodies (Fig. 1B). We assessed transcriptional activity using an ERE-tk-Luc reporter. Our findings revealed that *ESR1* fusions exhibited greater activity in the absence of estrogen compared to cells expressing ESR1-WT or ESR1-ΔCTD. Ligand stimulation with 1-nM E2 resulted in enhanced transcriptional activity of ESR1-WT compared to vehicle conditions (Fig. 1C). This response was not observed in cells expressing *ESR1* fusion proteins or ESR1-ΔCTD. Cotreatment with E2 (1 nM) and 100-nM of either the selective estrogen receptor degrader (SERD) fulvestrant (ICI182-780, ICI) or SERM tamoxifen (Tam) diminished ESR1-WT transcriptional activity compared to vehicle conditions; however, these treatments had no effect on *ESR1* fusion or ESR1-ΔCTD transcriptional activity (Fig. 1C). Our results indicate that *ESR1* fusions exhibit ligand-independent hyperactivation.

We next stably expressed the fusion proteins in a breast cancer cell line (C4-12) that was derived from MCF7 cells but has low to absent endogenous ER, depletion that is subject to passage numbers and culturing (Fig. 1D) (28). Interestingly, endogenous ER was consistently induced in cells stably coexpressing *ESR1* fusion proteins (see Fig. 1D). RT-qPCR with primers targeting the *ESR1* C terminal revealed a significant increase in *ESR1* mRNA in cells expressing ESR1-WT, a small increase in cells expressing ESR1-LPP, but no increase in cells expressing ESR1-ΔCTD, ESR1-DAB2, ESR1-SOX9, and ESR1-YAP1 (Supplementary Fig. S2) (50). In an ERE-luciferase reporter assay, the stable expression of the ESR1-WT plasmid led to an E2 response that was blocked by fulvestrant and tamoxifen (Fig. 1E). ESR1-ΔCTD exhibited minimal activity irrespective of treatment. ESR1-DAB2 and ESR1-SOX9 demonstrated hyperactivity in the absence of E2 and had a minimal response to E2 and ICI/Tam (see Fig. 1E). ESR1-LPP and ESR1-YAP1 did not demonstrate ligand-independent or endocrine-resistant activity, mimicking ESR1-WT (see Fig. 1E).

**Figure 2.**
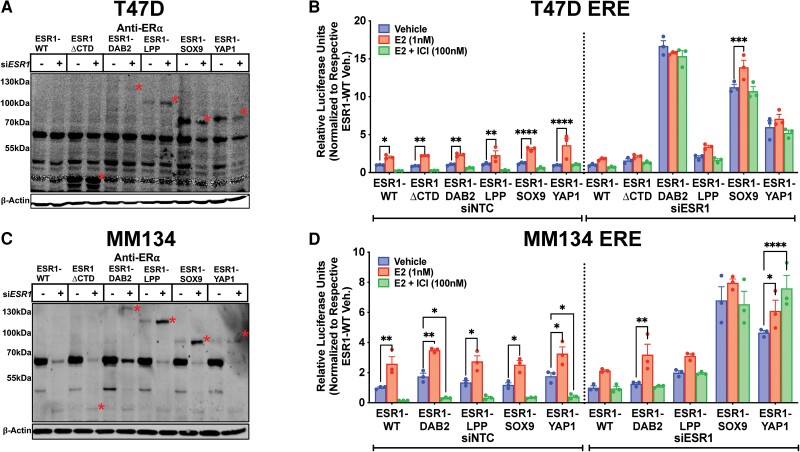
Increased fusion hyperactivity in T47D and MDA-MB-134-VI cells deplete of endogenous estrogen receptor (ER). A, Anti-ER immunoblot of transient *ESR1* small interfering RNA (siRNA) or scramble siRNA transfection in T47D cells stably overexpressing ER constructs. B, ER response element (ERE)-luciferase reporter assay in T47D cells stably expressing ER constructs transfected with nontargeting control (NTC) siRNA or siRNA targeting endogenous ER (siESR1). Raw luminescence values normalized to internal control, renilla luminescence, and to the representative siNTC- or siESR1-transfected ESR1-WT vehicle condition. Representative experiment shown with bar graph equivalent to readings ± SEM, n = 2. C, Anti-ER immunoblot of transient *ESR1* siRNA or scramble siRNA transfection in MDA-MB-134-VI (MM134) cells stably overexpressing ER constructs. Construct proteins denoted by red asterisks in all blots. β-Actin serves as loading control in all blots. D, ERE-luciferase reporter assay in MM134 cells stably expressing ER constructs transfected with NTC or siESR1. Normalization and control as above. Representative experiment shown with bar graph equivalent to readings ± SEM, n = 2. Note no ESR1-ΔCTD in MM134 ERE results. Two-way analysis of variance with post hoc Dunnett's multiple comparisons test showing enrichment in comparison to intraconstruct vehicle treatment in each assay. *P less than or equal to .05; **P less than or equal to .01; ***P less than or equal to .001; ****P less than .0001.

We sought to evaluate the effect of fusions in IDC-NST and ILC cell line models, T47D and MDA-MB-134-VI (MM134), respectively. To minimize interference from endogenously expressed ER, we knocked down ER using a siRNA targeting the C terminus of *ESR1*. Transient expression of siESR1 effectively reduced levels of endogenous ER in the T47D cell line, which was most pronounced with transient coexpression of ESR1-SOX9 and ESR1-YAP1 constructs (Fig. 2A). There was a slight decrease in immunoblot protein expression of ESR1-SOX9 and ESR1-YAP1. This reduction was not reproduced, both at the protein and mRNA level, when ESR1-SOX9 and ESR1-YAP1 cell lines were transiently transfected with the siRNA at higher molar concentrations (Supplementary Fig. S3) (50). In an ERE-luciferase reporter assay, *ESR1* fusions (ESR1-DAB2, ESR1-SOX9, and ESR1-YAP1) demonstrated more pronounced ligand-independent activity after the reduction of endogenous ER (displayed results are relative to ESR1-WT cells treated with siESR1 or control siRNA) (Fig. 2B). The efficient knockdown of endogenous ER in MDA-MB-134-VI was correlated to an increase in fusion protein expression levels, which may be potentially secondary to a compensatory adaptation mechanism or an artifact of short-term knockdown of endogenous ER (Fig. 2C). There was a ligand-independent, endocrine-resistant, hyperactive phenotype in the ESR1-SOX9 and ESR1-YAP1 MDA-MB-134-VI siRNA transfected cells, but not the ESR1-DAB2 transient cell line (Fig. 2D).

### Establishment of Stable Expression Models for Estrogen Receptor Fusions in T47D and MDA-MB-134-VI Cell Lines With Concurrent Endogenous Estrogen Receptor Knockdown

**Figure 3.**
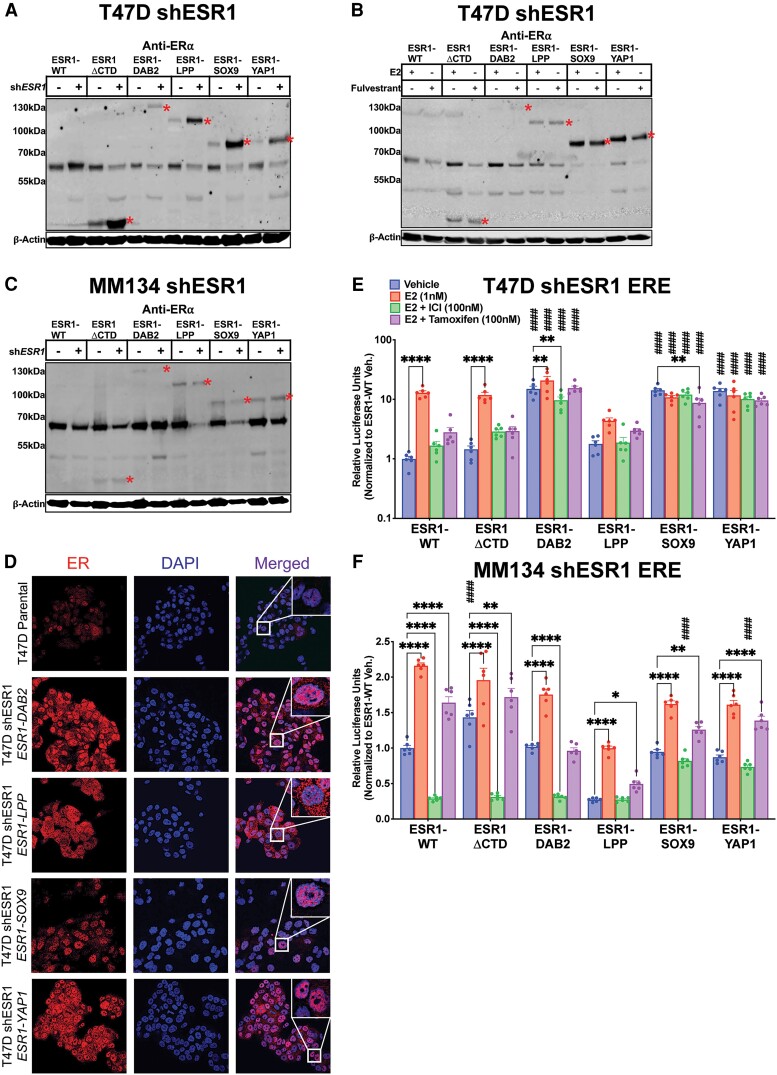
Stable estrogen receptor (ER) fusion overexpression in T47D and MDA-MB-134-VI endogenous ER-depleted cellular contexts. A, Anti-ER immunoblot of stably overexpressed ER constructs in T47D cell line stably infected with shESR1. B, Anti-ER immunoblot of stable T47D shESR1 cells treated in the presence of 1-nM 17β-estradiol (E2) or 100-nM fulvestrant. C, Anti-ER immunoblot of stably overexpressed ER constructs in MM134 cell line stably infected with shESR1. Construct proteins denoted by red asterisks in all blots. β-Actin serves as loading control in all blots. D, T47D parental and stable shESR1 cells stabling expressing plasmid constructs stained with anti-ER (red) and DAPI (4′,6-diamidino-2-phenylindole; blue) and imaged by confocal microscopy with a 40× objective. Merge represents overlay of anti-ER and DAPI staining. Representative experimental images, n = 2. E, ERE-luciferase reporter assays in stable T47D shESR1 cells and F, MM134 shESR1 cells expressing control and fusion constructs. Raw luminescence values normalized to internal control renilla luminescence and to the corresponding ESR1-WT vehicle condition luminescence readings. Representative experiment shown with bar graph equivalent to readings ± SEM, n = 3 for each experiment. Two-way analysis of variance with post hoc Dunnett's multiple comparisons test showing enrichment in comparison to corresponding ESR1-WT treatment (#) or to intraconstruct vehicle treatment (*) in each assay. */#*P* less than or equal to .05; **/##*P* less than or equal to .01; ***/###*P* less than or equal to .001; ****/####*P* less than .0001.

We next stably expressed ER fusions and controls through lentiviral infection of T47D and MDA-MB-134-VI cell lines. To eliminate the interference of endogenous ER, we reduced endogenous ER levels using shRNA targeting the 3′ UTR of *ESR1*. Knockdown of endogenous ER in IDC-NST T47D cells correlated with increased levels of *ESR1* fusion proteins (Fig. 3A), potentially secondary to increased cellular machinery availability for fusion stability. There was a less appreciable increase in ESR1-ΔCTD, ESR1-SOX9, and ESR1-YAP1 protein expression when using an additional shRNA, shESR1.2 (Supplementary Fig. S4A) (50). Treating the stable T47D cell lines with 100-nM fulvestrant resulted in degradation of residual endogenous ER protein expression and ESR1-WT. This treatment had little to no effect on ER fusion levels (Fig. 3B), indicating that activity of ER fusions exposed to E2, or endocrine therapies, is not confounded by changes in protein level. Compared to the transient ER knockdown experiment, MDA-MB-134-VI–infected shESR1 cells did not have a corresponding increase in ER fusion expression (Fig. 3C). Of note, ESR1-DAB2 endogenous ER knockdown was not as robust as compared to cells transiently transfect with siESR1 (Fig. 2C). Similarly, MDA-MB-134-VI cell lines had a less appreciable decrease in endogenous ER when stably transfecting cells with shESR1.2 (Supplementary Fig. S4B) (50).

Before investigating phenotypes, we performed immunofluorescence to examine the localization of ER fusions in comparison to parental T47D cell lines, serving as a control for nuclear endogenous ER (Fig. 3D). Among the ER fusions, ESR1-DAB2, ESR1-SOX9, and ESR1-YAP1 demonstrated nuclear localization whereas ESR1-LPP exhibited both nuclear and cytoplasmic ER staining (Fig. 3D). ESR1-DAB2, ESR1-SOX9, and ESR1-YAP1 demonstrated robust ligand-independent ERE hyperactivity in the IDC-NST cell line T47D shESR1 (Fig. 3E), whereas in MDA-MB-134-VI shESR1, hyperactivity was observed only for ESR1-SOX9 and ESR1-YAP1 under the fulvestrant treatment conditions (Fig. 3F). MDA-MB-134-VI shESR1 ESR1-DAB2 ERE activity did not reveal a ligand-independent or endocrine-resistant phenotype that was observed in the T47D shESR1 cells, mirroring the results from transient ER knockdown irrespective of the poor shESR1 endogenous ER knockdown (see Fig. 2).

### Distinct and Overlapping Estrogen Response Signatures Revealed by Estrogen Receptor Fusion Transcriptomic Profiling in T47D Short Hairpin *ESR1* Cells

**Figure 4.**
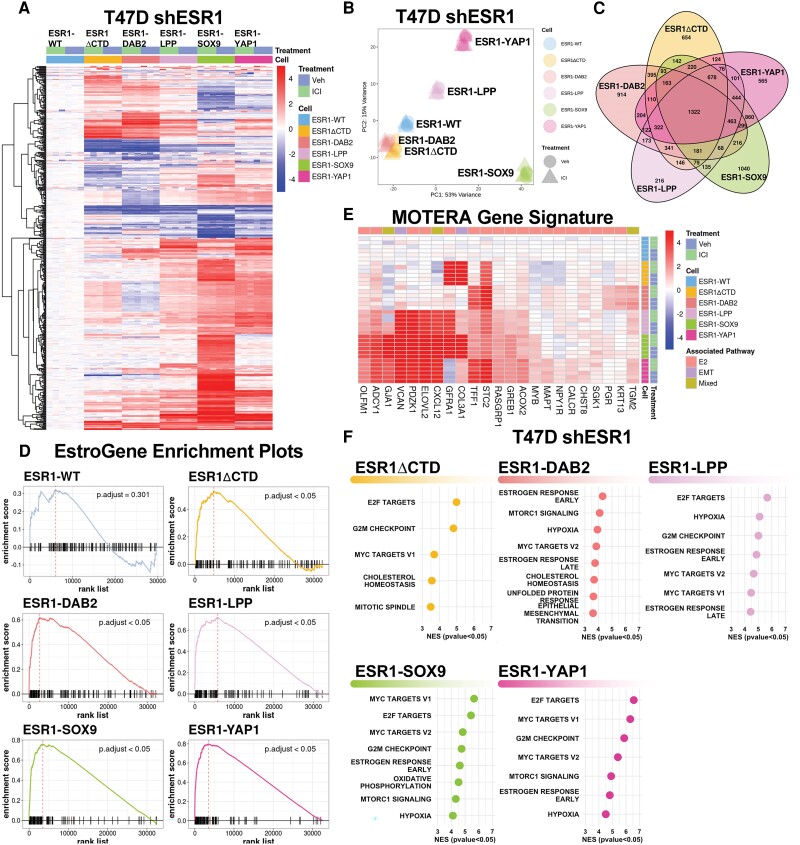
Transcriptomic profiles of estrogen receptor (ER) fusions in ER-depleted IDC-NST are enriched in estrogen-driven pathways. A, Heat map of the top 500 differentially expressed genes between vehicle and 100-nM ICI-treated cells with biased hierarchical clustering of columns. Data normalized to ESR1-WT for relative upregulation and downregulation. B, Principal component analysis (PCA) of T47D shESR1 ER constructs in the presence or absence of 100-nM ICI treatment. C, Venn diagram plot of ER construct differentially expressed genes (DEGs). DEGs generated between ESR1-WT and each ER construct (*P* < .05; FC > 0). D, Enrichment plots for ER construct DEG lists using modified T47D-specific EstroGene signature. ESR1-WT DEGs between treatment groups used as negative control. E, Heat map of the 24-gene signature, MOTERA [23]. Data normalized to ESR1-WT for relative upregulation and downregulation. F, Gene set enrichment analysis (GSEA) dot plots of fusion cell lines using the DEGs between each construct and ESR1-WT. Size of dot has no significance. X-axis denotes normalized gene enrichment scores (NES). All pathways plotted have NES *P* less than .05. DEGs generated between ESR1-WT and each ER construct (*P* < .05; FC > 0).

To evaluate both unique and shared activities of each fusion, we conducted RNAseq on the T47D shESR1 cell lines. These cells were subjected to treatment with either vehicle or 100-nM fulvestrant. This approach enabled us to assess potential residual endogenous ER activity and the effect of endocrine therapy on cells expressing ESR1-WT. Clustering of the top 500 DEGs revealed 3 primary groupings: (1) ESR1-WT, ESR1-ΔCTD, and ESR1-DAB2; (2) ESR1-LPP and ESR1-YAP1; and (3) ESR1-SOX9 (Fig. 4A). ESR1-WT subclustered independently from ESR1-ΔCTD, indicating ligand-independent activity of the activation function 1 (AF1) domain. Each construct clustered together irrespective of treatment condition. This was further confirmed by PCA (Fig. 4B), suggesting that fulvestrant had a minimal effect on transcriptional activity and that there was minimal response from endogenous ER given cells were hormone deprived prior to treatment. Thus, for subsequent analyses to determine DEG and pathway enrichment, the treatment groups (vehicle and fulvestrant) were combined as one entity for each cell line. There was no statistically significant difference in *ESR1* mRNA expression between cell lines expressing the different *ESR1* constructs (Supplementary Fig. S5) (50). ESR1-SOX9 and ESR1-YAP shared the most upregulated DEGs as predicted by global unbiased hierarchical clustering. A total of 463 shared DEGs were enriched in the 4 ER fusions cell lines compared to both ESR1-ΔCTD and ESR1-WT (Fig. 4C).

To assess the effect of ER fusions on the transcriptome, we compared DEGs to previously reported estrogen response signatures. Our laboratory has recently established EstroGene, a database containing publicly available RNAseq, ChIP-seq, and ATACseq data in breast cancer cell lines treated with vehicle or estradiol at various dosages and durations (39). From this data set, we selected the top 10th percentile of E2-regulated genes in T47D cells (n = 127) to serve as an estrogen response signature. ESR1-ΔCTD DEGs and all ER fusion DEGs were significantly enriched in the modified T47D-specific estrogen response signature compared to the DEGs associated with ESR1-WT (Fig. 4D). As expected, the ESR1-WT DEGs were not enriched given the absence of E2 and treatment with fulvestrant. We also sought to examine the signature Gou et al (27) developed to determine ER fusion activity, MOTERA (Mutant or Translocated Estrogen Receptor Alpha). Supervised clustering revealed the regulation of genes in the MOTERA in each of the ER fusions in comparison to ESR1-WT (Fig. 4E).

To better understand the contribution of the ER fusion partner to the chimeric protein function, we performed GSEA using the IndepthPathway package (37). In brief, genome-wide differential gene expression levels between *ESR1* fusions and ESR1-WT are used as a weighted gene list for pathway enrichment analysis using the Hallmark canonical pathway gene sets (51). The top 50 upregulated or downregulated pathways in *ESR1* fusion models are identified and then disambiguated based on the degree of overlap between pathway gene sets to reveal unique pathways (52). Our results revealed that in all models expressing fusion constructs, the “estrogen response early” pathway was significantly upregulated. The oncogenic “MYC targets V1” and “MYC targets V2” were upregulated in all the ligand-depleted transcriptomes of the ER fusions that lack LBD (including ESR1-ΔCTD). In addition, ESR1-DAB2, ESR1-SOX9, and ESR1-YAP1 models showed significant upregulation of the “mTORC1 signaling” pathway (Fig. 4F). All ER fusion models show upregulation of “hypoxia” pathways, whereas the “epithelial mesenchymal transition” signature was enriched only in the ESR1-DAB2 model. The overall similarity of these independent pathway enrichments derived from unique transcriptomes raises promise regarding potential shared pathways among fusion proteins irrespective of the 3′ partner gene.

Gap junction *GJA1* was previously found to be enriched in fusion-positive cells (27). Recent work by Li et al (12) uncovered an important role of gap junction proteins in *ESR1* point mutation (Y537S and D538G)-driven tumor progression. Gene expression in the ESR1-SOX9 cells were markedly enriched in the connexin gene family, suggesting a unique functionality to ESR1-SOX9 that remains to be further investigated (Supplementary Fig. S6) (50). In summary, transcriptomic profiling of T47D cells overexpressing ER fusion proteins demonstrated pathway enrichment shared between the fusions, as well as unique changes that might contribute to the varying levels of endocrine resistance activity.

### Cistromic Profiling Reveals Unique Transcriptional Regulation by Estrogen Receptor Fusions Compared to ESR1-WT

**Figure 5.**
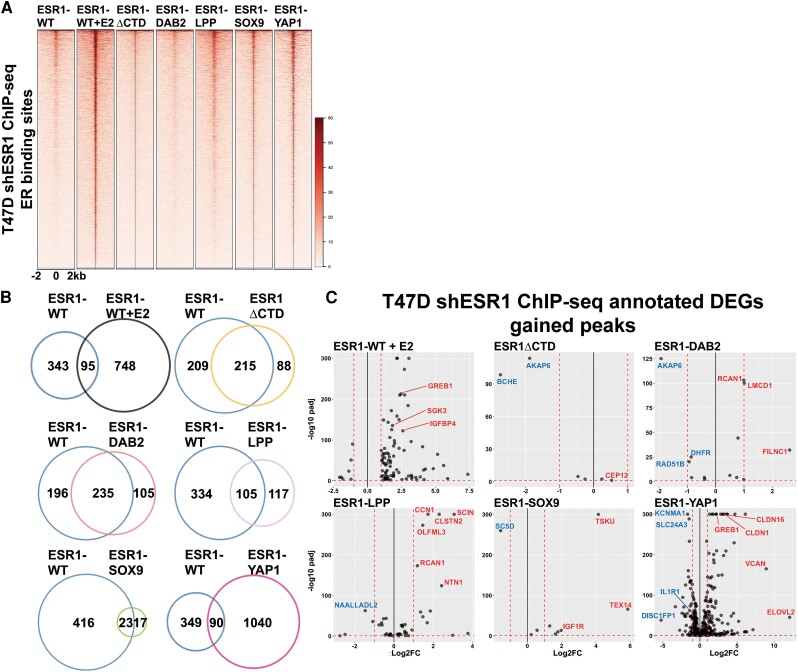
Fusion cistromes reveal direct fusion transcriptomic functionality. A, Heat map of estrogen receptor (ER) binding intensities on T47D shESR1 HA chromatin immunoprecipitation sequencing (ChIP-seq)-detected sites in ESR1-WT and ER constructs. Displayed in horizontal window of ±2 kb from peak center. B, Venn diagrams displaying overlap of shared and unique genomic binding sites between ESR1-WT and ESR1-WT stimulated with 1-nM 17β-estradiol (E2) or ESR1-WT and ER constructs. C, Volcano plots of genes upregulated and downregulated both in transcriptomic and cistromic analysis. Select upregulated genes are highlighted in red and select downregulated genes are highlighted in blue.

To better understand ER fusions activity as transcription factors, ChIP-seq was performed using the HA tag present in all ER constructs. Hormone-deprived ESR1-WT cells treated with 1-nM E2 for 45 minutes served as a control for the E2 induced ER cistrome; all other cells were examined under vehicle conditions, that is, without E2. ESR1-LPP, ESR1-SOX9, and ESR1-YAP1 displayed enriched binding at the well-known E2-regulated ER binding sites in *GREB1* and *IGFBP4* (Supplementary Fig. S7) (50). Furthermore, ligand-depleted ESR1-LPP, ESR1-SOX9, and ESR1-YAP1 had enhanced ChIP-seq signals at ER binding sites, at an intensity similar to that of the E2-treated ESR1-WT cell cistrome (Fig. 5A). On comparison to previously published T47D ESR1-YAP1 ChIP-seq, there was a 40% overlap in binding peaks with our data despite varied ChIP-seq methods (Supplementary Fig. S8) (50). To further investigate fusion-specific cistromes, the overlap of canonical ER binding sites was identified between each fusion protein and ESR1-WT. As expected, ESR1-WT cells treated with E2 showed an enhanced number of unique ER binding regions compared to vehicle-treated ESR1-WT cells (Fig. 5B). ESR1-ΔCTD and ESR1-DAB2 shared more ER binding regions with ESR1-WT compared to new unique binding sites. ESR1-LPP shared 105 binding regions with ESR1-WT but was also bound to 117 unique regions. ESR1-SOX9 had a strong overlap with ESR1-WT (57.5%) and relatively few unique ER binding sites; however, of the unique ESR1-SOX9 binding sites, 94% contained ERE motifs (Supplementary Tables S1 and S2) (50). ESR1-YAP1 had 1040 unique ER binding sites compared to ESR1-WT; 23% of the unique binding regions harbored ERE motifs, suggesting a more promiscuous binding to ER-associated regions irrespective of ERE.

The unique binding peaks of each *ESR1* fusion compared to ESR1-WT were then integrated with their associative transcriptomes to develop a list of DEGs that could help explain fusion transcriptional regulation at gene loci that may contribute to treatment-resistance phenotypes (Supplementary Table S3) (50). The ESR1-DAB2 cistromic-transcriptomic overlap revealed enrichment of *RCAN1*, which has an isoform (*RCAN1.4*) that has been associated with tumor suppression in one study, as well as an upstream negative regulator of Myc-1, *FILNC1* (Fig. 5C) (53, 54). ESR1-LPP was also found to have enhanced binding and subsequent transcription of *RCAN1*. In addition, however, ESR1-LPP was enriched in transcription of oncogenic *CCN1* and *NTN1* (55, 56). ESR1-SOX9 cistromic-transcriptome revealed downregulation of *SC5D,* which is a positive prognostic indicator of neoadjuvant chemotherapy response (57). The cistromic-transcriptomic overlap also revealed enrichment of *IGF1R* in ESR1-SOX9–expressing cells. Our research group has studied the role of *IGF1R* in the context of different molecular and histological subtypes of breast cancer (58-62). The enrichment of the gene provides a potential novel axis to target ESR1-SOX9–positive tumors. ESR1-YAP1 had the most congruence between cistromic and transcriptomic analyses, suggesting direct transcriptional activity. Analysis revealed an upregulation of a chemoresistant biomarker, *CLDN16* (63). In addition, another claudin family member, *CLDN1*, was upregulated in ESR1-YAP1 cells. *CLDN1* has been described as downregulated in luminal breast cancer, but upregulated in aggressive basal-like subtypes, which may potentially suggest subtype switching for cells expressing ESR1-YAP1 or other ER fusions (64). ESR1-YAP1 cistromic-transcriptomic overlap also revealed enrichment of *ELOVL2,* a tumor suppressor in tamoxifen-resistant settings, which emphasizes the need for further investigation in how tumor suppressors may be functioning in fusion-dependent contexts (65). ESR1-YAP1 was found to have enhanced peak enrichment at the *GREB1* and *VCAN* locus, supporting the fusion's functionality in estrogen-mediated and migratory pathways. Given the similarity in pathway enrichment between the fusion constructs, the differences in fusion cistromes implies a diversity in upstream genomic regulation that ultimately results in shared cellular activity.

### The Contribution of Estrogen Receptor Fusion Proteins to Oncogenic Phenotypes Are Assay Dependent and Cell Line Specific.

**Figure 6.**
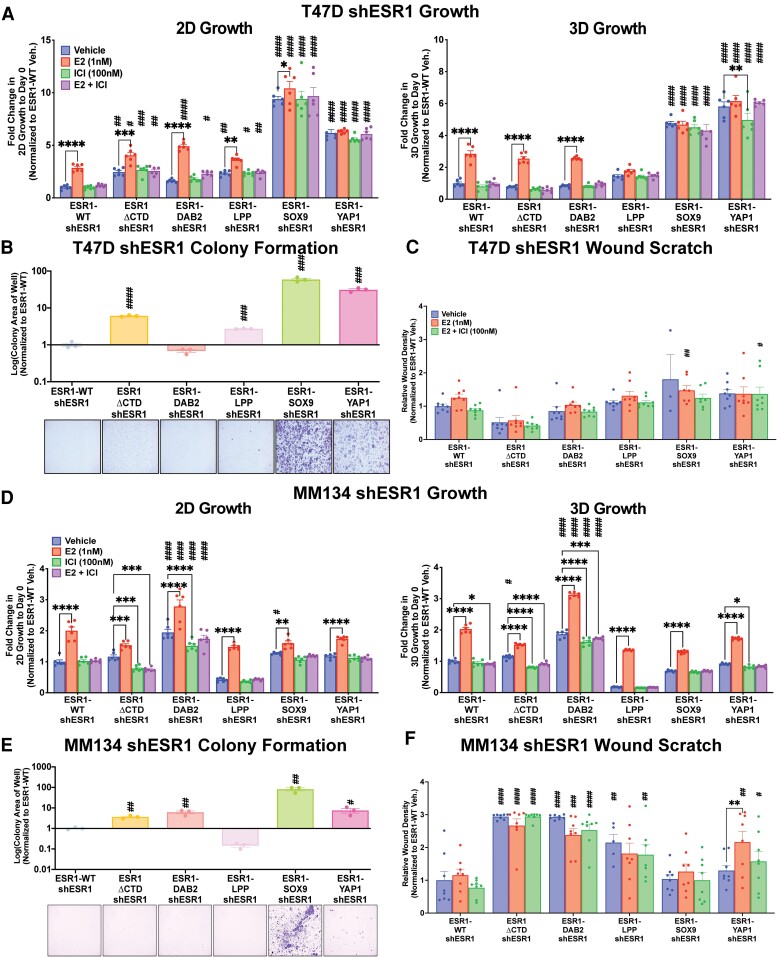
Cellular context-dependent fusion enrichment in metastatic properties. Two-day (2D) and 3-day (3D) growth in A, T47D and D, MM134 shESR1 cell lines. Cell viability was quantified on days 0 and 9 (T47D) or 4 (MM134) with CellTiter-Glo assay, and data were normalized to day 0 quantification. Representative experiment shown with readings ± SEM, n = 3 (each with 6 technical replicates). B, T47D shESR1 and E, MM134 shESR1 colony formation assays. Cells were seeded at a low cell density and stained with 0.5% crystal violet at approximately 2 weeks (T47D) or 3.5 weeks (MM134). Representative graphs generated from ImageJ particle analyzer plugin quantification and representative images are shown, n = 3 (each with 3 technical replicates, SEM error bars). Unpaired *t* test performed between each construct and ESR1-WT. Wound scratch healing assay of C, T47D shESR1 and F, MM134 shESR1 cells in the presence of antiproliferation treatment with mitomycin C. Bar graphs generated from IncuCyte automated program to calculate relative wound density after a 72-hour time period, n = 3 (each with 8 technical replicates, SEM error bars). Two-way analysis of variance with post hoc Dunnett's multiple comparisons test showing enrichment in comparison to corresponding ESR1-WT treatment (#) or to intraconstruct vehicle treatment (*) in growth and migration assays. */#*P* less than or equal to .05; **/##*P* less than or equal to .01; ***/###*P* less than or equal to .001; ****/####*P* less than .0001.

To understand fusion contribution to endocrine resistance and metastatic phenotypes, we examined growth, cell survival, and cell migration in the IDC-NST– and ILC-stable ER fusion-overexpressing cell line models. Consistent with ERE hyperactivity, T47D shESR1 ESR1-SOX9 and ESR1-YAP1 cell lines displayed enhanced proliferation compared to ESR1-WT, irrespective of treatment condition (Fig. 6A). ESR1-LPP had a more consistent ligand-independent, endocrine-resistant growth pattern both in 2D and 3D conditions compared to ESR1-DAB2 (see Fig. 6A). Consistent with the growth phenotype, T47D shESR1 ESR1-LPP, ESR1-SOX9, and ESR1-YAP1 stable cell lines displayed enhanced colony formation in hormone-deprived conditions and at low cellular densities compared to ESR1-WT (Fig. 6B). ESR1-DAB2, analogous to the growth assay, did not have a robust colony formation phenotype (see Fig. 6B). We next assessed migration of the T47D shESR1 cells employing a wound scratch assay. Although not significant, at the 72-hour time point post scratch creation, there was a consistent trend in wound closure in the ESR1-SOX9 and ESR1-YAP1 cell line models (Fig. 6C). ESR1-DAB2 and ESR1-LPP cell migration was similar to that of the ESR1-WT cell line.

**Figure 7.**
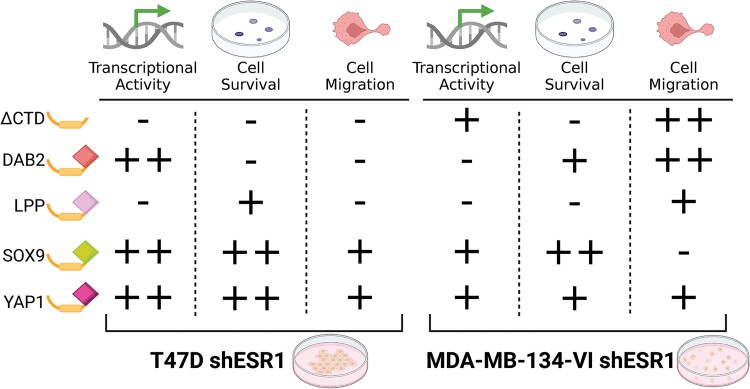
Estrogen receptor (ER) fusion phenotype summary. Schematic of ER fusions and corresponding oncogenic activity in T47D and MM134 cell lines. Plus sign indicates activity. Minus sign signifies no appreciable activity. Activity is estimated in comparison to ESR1-WT in ligand-independent and endocrine-resistant conditions. Created with BioRender.com.

To further characterize the fusions in an ILC cell line model, we performed the same assays in the MDA-MB-134-VI shESR1 cell lines, which stably expressed the ER constructs. Endocrine-resistant growth was not evident in the MDA-MB-134-VI shESR1 cells overexpressing the ER fusions, except for ESR1-DAB2 cells (Fig. 6D). Although MDA-MB-134-VI cells overexpressing ESR1-SOX9 and ESR1-YAP1 did not display enhanced 2D growth phenotypes, they showed significantly enhanced colony formation compared to ESR1-WT cells (Fig. 6E). We also observed more robust colony formation (see Fig. 6E) as well as an increased migratory phenotype in ESR1-DAB2 MDA-MB-134-VI shESR1 cells (Fig. 6F). ESR1-ΔCTD in the MDA-MB-134-VI shESR1 cells also presented a higher migratory phenotype than ESR1-WT (see Fig. 6F). The stark differences between the phenotypes observed in the IDC-NST T47D and ILC MDA-MB-134-VI fusion cell lines requires further investigation into context-dependent mechanisms of action of the *ESR1* fusion protein (Fig. 7).

## Discussion

Endocrine resistance affects a third of primary ER-positive breast cancer cases and the majority of metastatic disease (66). While ER point mutations (eg, Y537S and D538G) respond to estrogen and antiestrogens, albeit weaker than WT ER, ER fusion proteins lack a hormone-binding domain and thus represent a mechanism of pure ligand-independence and endocrine resistance. Herein, we report that 4 ER fusion proteins are functionally active in HEK293 cells irrespective of E2 or endocrine therapy stimulation. These results held true in C4-12 breast cancer cells that are devoid of endogenous ER. In IDC-NST T47D and ILC MDA-MB-134-VI cell line models, transient and stable knockdown of endogenous ER enabled dissection of fusion-specific phenotypes. The robust ERE response in T47D shESR1 cells expressing ESR1-DAB2, ESR1-SOX9, or ESR1-YAP1 differed from that observed in the ILC cell line, suggesting a potential histological subtype influence on fusion function; however, additional cell lines need to be tested. Through RNAseq, we report that T47D shESR1 fusion transcriptomes subcluster from ESR1-WT in E2-depleted and fulvestrant-treated conditions. All fusion transcriptomes shared estrogen and oncogenic pathway enrichment, with the transcriptionally active fusions (as measured in the ERE-luciferase reporter assays), ESR1-SOX9 and ESR1-YAP1, demonstrating the greatest overlap in DEGs. The direct and indirect activity of fusions varied, with fusion partners that were transcription factors harboring the most activity. Finally, we present our findings regarding the phenotypes of these fusions in the IDC-NST and ILC shESR1 cell line models.

Since the discovery of an ESR1-YAP1 fusion in 2013 (26), various *ESR1* exon 6 fusion events have been identified; however, few have been comprehensively studied. Lei et al (49) reported increased transcriptional activity of ESR1-YAP1 and ESR1-PCDH11X as evidence of ERE binding, and in vivo dose-dependent growth inhibition with palbociclib. While Lei et al (49) reported that *ESR1* fusions do not heterodimerize with WT ER, endogenous ER presumably competes for DNA binding sites or squelches ER coactivators, thus complicating interpretation of fusion-driven cellular activity. Indeed, using cells with depletion of endogenous ER, we observed more robust ER fusion-mediated phenotypes that are independent of the endogenous ER influence. An important limitation of the study is the risk of off-target effects with the introduction of the shRNA in cell lines already transfected with chimeric plasmids. Targeting of the *ESR1* 3′ UTR with an additional shESR1 (shESR1.2) in the T47D cells resulted in a less appreciable associative change in fusion protein expression, suggesting that targeting the 3′ UTR does not alter the chimeric ER plasmid expression.

In studying *ESR1* fusions, Gou et al (27) developed a fusion activity transcriptomic signature, MOTERA, which is composed mostly of estrogen-responsive genes in addition to some epithelial-to-mesenchymal transition (EMT) genes. We examined the expression of a cohort of upregulated E2-responsive genes derived from T47D RNAseq data that are publicly available through the EstroGene database (39). Gene expression of cells overexpressing our 4 fusion constructs and the truncated fusion protein were enriched in a T47D-specific EstroGene signature, emphasizing that ER fusions retain components of the canonical ER regulome. Consistent with this, all fusions showed MOTERA signature gene enrichment. The enrichment of estrogen-regulated genes in cells expressing ESR1-ΔCTD is notable and presumably reflects the ability of AF1 to activate transcription. This is particularly interesting in MDA-MB-134-VI cells given the colony formation and migration phenotypes of ESR1-ΔCTD.

The diverse nature of ER fusion 3′ partners are hypothesized to influence unique fusion-specific transcriptomes. However, we found similarity in enriched pathways among the hormone-deprived fusion transcriptomes, including upregulation of proliferative and EMT-related pathways. MYC (v-myc avian myelocytomatosis viral oncogene homologue) targets is a gene set enriched in all ligand-depleted *ESR1* chimeric cell lines compared to ESR1-WT, suggesting regulation by AF1. The MYC pathway has been implicated in breast cancer progression and is associated with poor prognosis (67). Furthermore, MYC signaling has been described in antiestrogen resistance and distant relapse of ER-positive disease (68). The consistency in the upregulation of the MYC pathway, even after disambiguation, shows a promising overlap between the otherwise distinct phenotypic responses from each fusion.

We report findings of ER fusion activity in an ILC cell line. ILC is one of the most common histologic subtypes of breast cancer and is predominantly ER positive. ER point mutations developed in metastatic disease do not differ in type nor prevalence between IDC-NST and ILC breast tumors; therefore it is imperative to study fusions in both representative disease models (69). We reported an increased sensitivity to molecular chaperone inhibition in MDA-MB-134-VI cells compared to T47D cells, suggesting a potential mechanism of fusion or downstream chaperone-mediated transcriptome stabilization, particularly in a setting of elevated proteomic stress (70). Our present work suggests that the distinct molecular landscape of an ILC cell line influences fusion behavior. For example, ESR1-LPP demonstrated a migratory phenotype in MDA-MB-134-VI cells compared to a lack of activity in the IDC-NST cell line model. Similarly, a reproducible significant growth enrichment was observed in ILC ESR1-DAB2 cells compared to the ESR1-WT cell line. Interestingly, Gou et al (27) described differences in growth of ESR1-DAB2–expressing cells between 2 IDC-NST cell lines but ultimately described the fusion as inactive in their MOTERA signature. A limitation of the study revolves around the poor endogenous ER knockdown in MDA-MB-134-VI shESR1 cells expressing ESR1-DAB2. Our hypothesis regarding endogenous ER interference with fusion activity may not be as pronounced or evident in MDA-MB-134-VI ESR1-DAB2 cells, Importantly, ESR1-DAB2 ERE-luciferase reporter activity was not significantly different between the more efficient siESR1 transfection and the less efficient shESR1 infection. The enhanced growth, survival, and migration in MDA-MB-134-VI shESR1 ESR1-DAB2 cells may be irrespective of endogenous ER expression, but further studies are required to better understand the expression-function relationship between the chimeric ESR1-DAB2 and WT endogenous protein. An additional limitation in the study is the lack of MDA-MB-134-VI shESR1 transcriptomic and cistromic analyses to (1) support experimental transcriptional activity and phenotypes, (2) decipher mechanisms of action, and (3) compare to the T47D shESR1 RNAseq and ChIP-seq data. Further investigation into subtype-specific *ESR1* chimeric protein functionality in both in vitro and in vivo models is warranted.

The transcriptome of ESR1-LPP was surprisingly more similar to ESR1-SOX9 and ESR1-YAP1 than hypothesized based on phenotypic assays in which ESR1-LPP showed minimal activity. However, ESR1-LPP was found to be both nuclear and cytoplasmic; thus, the fusion may be involved in regulating extranuclear functions that may ultimately converge on similar global transcriptomic profiling as ESR1-SOX9 and ESR1-YAP1. Another unexpected finding was the enrichment of collagen type III alpha 1 chain (*COL3A1*) in ESR1-ΔCTD–positive cells. *COL3A1* is a member of the EMT regulome that is associated with poor prognosis in ER-positive breast cancer (71). In T47D cells, ESR1-ΔCTD displayed no enhanced migration; however, in MDA-MB-134-VI cells, there was significantly elevated motility. Whether *COL3A1* influences an enhanced MDA-MB-134-VI ESR1-ΔCTD phenotypic response requires further experimentation. ESR1-SOX9 was additionally enriched in *COL3A1* expression as well as oxidative phosphorylation and cell adhesion pathway members. We have previously published on enhanced cell-cell adhesion networks in *ESR1* point mutant models that translated to increased circulating tumor cell clusters in patients with metastatic, endocrine-resistant disease (12). The similarity of heightened cell adhesion properties in *ESR1* fusions supports a potential avenue to targeting all functioning *ESR1* chimeras with disruption of the LBD. Finally, ERE motifs were enriched in ESR1-YAP1, consistent with previously reported data (49).

In our present study, we have also demonstrated congruent functional phenotypes to the current literature (27, 49). Specifically, we observed consistent growth, cell survival, and migration in T47D shESR1 cell lines expressing ESR1-SOX9 and ESR1-YAP1; notably, these fusions were consistently the most active. As previously described, ESR1-DAB2 displayed context dependent activity. DAB2-positive tumor-associated macrophages remodel the extracellular environment and are associated with overall poor clinical outcome in ILC (72). Although this phenomenon has been strictly reported in tumor-associated macrophages, there is the potential that the DAB2 fusion protein remodels the extracellular matrix in the higher stromal microenvironment of ILC cell lines. Consistent with this notion is ESR1-DAB2 cell line enrichment in EMT, which may enable enhanced phenotypes in the MDA-MB-134-VI cellular context. Surprisingly, ESR1-SOX9 did not have as robust phenotypes in growth and migration in the ILC MDA-MB-134-VI cells. Interestingly, cell survival was enriched in all T47D constructs besides ESR1-DAB2, which may contribute to the lack of upregulation in E2F target genes.

The role of the 3′ partner in fusion functionality remains an active research endeavor. Despite the enrichment of E2 signatures, the diverse functional profiles suggest a predominant influence from the translocated gene. In addition, the fusion models’ enrichment in the “mTORC1 signaling” pathway was not exhibited in ESR1-ΔCTD, suggesting a functional or stabilizing role of the 3′ partner, whereas MYC-related pathways may contribute to *ESR1* exons 1 to 6. Annotated cistrome DEGs revealed ESR1-YAP1–regulated transcription of the canonical YAP1 regulome, including fatty acid elongation and claudin family members, indicating a canonical influence of the YAP1 transcription coactivator on the fusion transcriptional activity. This may help explain the enhanced functional activity that we and others have reported in the ESR1-YAP1 model. Similarly, enhanced ESR1-SOX9 activity may be secondary to the 3′ partner's role as a transcription factor, however, potentially through a coactivating or stabilizing function as the ESR1-SOX9 cistrome was enriched in ERE-binding motifs. The selective affinity of ESR1-SOX9 for binding EREs in an E2-depleted context suggests a narrow, yet influential, regulation of ER-related gene regions.

Collectively, our findings augment the limited literature on ER fusion proteins in advanced treatment-resistant breast cancer. Critically, our work underscores the role of cellular context and phenotypic properties in influencing the actions of ER fusion proteins. Continued investigation of ER fusion proteins both in IDC-NST and ILC contexts may provide alternate treatment regimens in patients diagnosed with endocrine-resistant breast cancer, an acutely relevant endeavor in the setting of updated guidelines to identify ER mutants during therapeutic intervention (73).

## Data Availability

Original data generated and analyzed during this study are included in this published article or in the data repositories listed in “References.”

## Abbreviations

AF1: activation function 1
ChIP-seq: chromatin immunoprecipitation sequencing
DEGs: differentially expressed genes
E2: 17β-estradiol
EMT: epithelial-to-mesenchymal transition
ER: estrogen receptor
ERE: estrogen receptor response element
*ESR1*: estrogen receptor
GSEA: gene set enrichment analysis
HA: human influenza hemagglutinin
IDC: invasive ductal carcinoma
ILC: invasive lobular carcinoma
LBD: ligand-binding domain
mRNA: messenger RNA
NST: no-special type
PBS: phosphate-buffered saline
PCA: principal component analysis
qRT-PCR: quantitative real-time polymerase chain reaction
RNAseq: RNA sequencing
SERMs: selective estrogen receptor modulators
shRNA: short hairpin RNA
siRNA: small interfering RNA
UTR: untranslated region
WT: wild-type
